# Opposite effects of low-carbohydrate high-fat diet on metabolism in humans and mice

**DOI:** 10.1186/s12944-023-01956-3

**Published:** 2023-11-10

**Authors:** Lingli Cai, Xinyi Xia, Yunjie Gu, Lili Hu, Cheng Li, Xiaojing Ma, Jun Yin

**Affiliations:** 1 Department of Endocrinology and Metabolism, Shanghai Sixth People’s Hospital Affiliated to Shanghai Jiao Tong University School of Medicine, Shanghai Clinical Center for Diabetes, Shanghai Key Laboratory of Diabetes Mellitus, Shanghai Diabetes Institute, Shanghai, 200233 China; 2 Department of Endocrinology and Metabolism, Shanghai Eighth People’s Hospital, Shanghai, 200235 China

**Keywords:** Ketogenic diet, High-fat diet, Appetite, Food preference, Energy intake, Homeostatic mechanism

## Abstract

**Background:**

Low-carbohydrate diet (LCD) is effective for weight loss and glycaemic control in humans. Here, the study aimed to explore the effects of LCD/high-fat diet (HFD) in both humans and mice.

**Methods:**

Twenty-two overweight or obese participants received LCD for 3 weeks. Based on carbohydrate intake > 10% or ≤ 10% of calories, the participants were divided into moderate LCD (MLCD) and very LCD (VLCD) groups. The participants completed a 10-question food preference survey. Meanwhile, C57BL/6J mice were assigned to five groups: chow diet (CD, 10% fat), HFD with 60%, 70%, and 75% fat from cocoa butter (HFD-C), and HFD with 60% fat from lard (HFD-L) and fed for 24 weeks. Eight mice were acclimatised for the food-choice test.

**Results:**

LCD decreased the total energy intake in humans. The VLCD group showed greater weight loss and better glycaemic control than the MLCD group. A food preference survey showed that 65% of participants tended to choose high-carbohydrate foods. In mice, HFD resulted in energy overconsumption, obesity, and metabolic disorders. When CD and HFD-L were administered simultaneously, mice rarely consumed CD. In the HFD-C groups, the energy intake and body weight increased with increasing dietary fat content. Compared with the HFD-C group, the HFD-L group consumed more energy and had poorer metabolism.

**Conclusions:**

Lower carbohydrate intake contributed to lower energy intake and improved metabolism in humans. In mice, diets with a higher proportion of fat become more attractive and obesogenic by fixing the fat sources. Since the mice preferred lard to cocoa butter, lard induced excess energy intake and poorer metabolism. Different food preferences may be the underlying mechanism behind the opposite effects of the LCD/HFD in humans and mice.

**Trial registration:**

The clinical trial was registered with the Chinese Clinical Trial Registry (www.chictr.org.cn). The registration number is ChiCTR1800016786. All participants provided written informed consent prior to enrolment.

**Supplementary Information:**

The online version contains supplementary material available at 10.1186/s12944-023-01956-3.

## Background

The prevalence of obesity in adults has reached 12%, and approximately 2.3 billion individuals worldwide are overweight or obese [1, 2]. Obesity stands as a significant risk factor for chronic noncommunicable diseases, including cardiovascular diseases, type 2 diabetes, hypertension, and cancer, imposing substantial health and socioeconomic burdens on society [3]. Although sustaining a healthy lifestyle for a long time can indeed be challenging, lifestyle interventions, particularly dietary strategies, consistently considered as the primary approach to managing obesity and various metabolic disorders before pharmaceutical interventions. Low-carbohydrate diets (LCDs) have garnered substantial interest due to their benefits in weight loss, glycaemic control, and reduction in serum triglyceride (TG) [4–7]. High carbohydrate consumption leads to hyperinsulinemia, a condition that fosters fat accumulation and exacerbates adiposity, especially in individuals with metabolic disorders such as obesity and diabetes. LCDs, on the other hand, restrict carbohydrate intake, rely on fat as the primary energy source, alleviate water-sodium retention, reduce insulin secretion, and facilitate fat mobilisation from adipose tissue, thus contributing to weight loss and metabolic improvement [8, 9].

In our prior clinical trial, we observed that an LCD improved glucose homeostasis, alleviated fatty liver disease, and reduced body weight [10]. These findings suggested its superior efficacy in promoting weight loss and regulating glucose homeostasis compared to short-term exercise. In the present study, the authors reassembled and reanalysed the data from the trial by dividing the participants who followed LCDs into two groups based on their carbohydrate intake: the moderate LCD (MLCD) group and the very LCD (VLCD) group. Notably, the fat intake in the VLCD group was approximately 60% of the total daily energy intake. The lower the proportion of carbohydrates, the better the metabolism (details of the analysis are provided in the subsequent section).

In humans, LCDs generally refer to carbohydrate intake < 130 g/day or less than 26% of the 2000 kcal/day [11]. When the carbohydrate intake drops to < 10% of the total energy consumed, it is also called a VLCD or ketogenic diet. A reduction in carbohydrate consumption is usually accompanied by an increase in dietary fat and, to a lesser extent, protein [12]. The macronutrient composition of a high-fat diet (HFD) used in animal experiments is similar to that of an LCD. In HFD for mice, fat typically constitutes 40–60% of the total energy and may include various fat sources such as lard, coconut oil, cocoa butter, soybean oil, and more. It’s worth noting that, unlike LCDs in humans, HFDs in mice are primarily employed to induce obesity and mimic a range of human metabolic disorders. When C57BL/6J mice are provided with HFD ad libitum, they tend to develop metabolic abnormalities, including obesity, hyperlipidemia, and hyperglycemia [13, 14]. Inspired by the results of the aforementioned clinical trial, which demonstrated a positive correlation between reduced carbohydrate intake and improved metabolism but lacked an in-depth mechanistic exploration, we wanted to apply similar dietary strategies to mice, aiming to replicate the findings observed in humans and delve into the underlying mechanisms driving these outcomes.

Lard and cocoa butter are two of the most commonly used sources of animal- and plant-derived fats, respectively, in HFD designed for mice. Lard consists of 45% saturated fatty acids (SFAs) [15], 45% monounsaturated fatty acids (MUFAs) [16] and approximately 9–10% polyunsaturated fatty acids (PUFAs) [17]. Cocoa butter mainly comprises 62.0% SFAs, 33.6% MUFAs, and 2.7% PUFAs [18]. Unlike vegetable oils such as soybean oil, both lard and cocoa butter can be processed into rod-like solids, which have a geometric shape similar to that of the standard chow diet (CD), even when their proportion is increased to 60% or more (cocoa butter only). Additionally, cocoa butter maintains its stability as a solid at room temperature due to its short plastic range. In contrast, products containing lard transition into a paste-like consistency at room temperature when the lard proportion exceeds 60%. Therefore, diets with 60%, 70%, and 75% cocoa butter and 60% lard were finally chosen, which were all stable with similar geometric shapes, to feed the mice.

Hence, based on our prior clinical trial, a post hoc analysis and animal experiments are conducted in this study to investigate the impacts of an LCD/HFD on metabolism in humans and mice.

## Methods

### Human study: study subjects and protocol

Informed consent was obtained from all the participants. This trial was approved by the Ethics Committee of Shanghai Jiao Tong University School of Medicine, Affiliated Sixth People’s Hospital. The trial was registered with the Chinese Clinical Trial Registry (ChiCTR1800016786). The inclusion criteria were as follows: age 18–40 years, BMI ≥24 kg/m^2^ and waist circumference ≥90 cm in male and ≥85 cm in female [19]. The exclusion criteria included a diagnosis or history of diabetes mellitus and recent participation in any weight loss interventions within 1 month before the study. A more detailed methodology has been previously described [10, 20]. In brief, 22 participants were instructed to go on an LCD (carbohydrate intake < 50 g/day was suggested) for 3 weeks with no restrictions imposed on calorie, fat, or protein intake. During the intervention period, participants were required to document their daily dietary intake and submit photographs of their meals to the investigators via WeChat, a widely used mobile social application in China. Furthermore, participants were required not to modify their other usual lifestyle or engage in additional physical exercise throughout the intervention. Based on their carbohydrate intake, the 22 participants were categorized into two groups after the intervention. Carbohydrate intake was > 10% and ≤ 10% of the total energy intake in the MLCD group (*n* = 9) and in the VLCD group (*n* = 13), respectively.

The first day of the intervention was designated as day 1, and the final day was designated as day 21. Anthropometric parameters were measured on day − 3 (three days before the intervention commenced) and day 22 (the day following the completion of the intervention). The participants were instructed to wear flash glucose monitors continuously, recording glucose values from day − 3 to day 24 (utilizing two sensors throughout the entire intervention). The participants reverted to their prior diet between days 22 and 24. The mean sensor glucose (MSG), standard deviations of sensor glucose (SDSG), coefficient of variation (CV), and largest amplitude of glycaemic excursion (LAGE) were calculated to assess daily glycaemic levels and fluctuations [21]. MSG is the mean glucose value that represents a simple summary of overall glycaemic control. SDSG and CV quantify the relative variability of glucose levels in relation to the mean, and LAGE represents the difference between the highest and lowest glucose values within a defined period, highlighting the largest fluctuations in glucose levels.

The participants completed a 10-question food preference survey questionnaire to gain insights into their dietary habits and investigate whether food preferences had an impact on metabolism. For each question, the participants were asked to choose their preferred dish from the two or three listed dishes. The dishes were either high-carbohydrate or high-fat foods, with at least 50% of their calories derived from carbohydrates or fat, respectively. To ensure comparability, the dishes presented for the same question shared similar appearances and raw materials. Option for high-carbohydrate foods was scored as 1 and − 1 of option for high-fat foods. The cumulative score was then calculated by summing the scores for all the questions. A total score > 0 indicated a preference for high-carbohydrate foods, while a score < 0 indicated a preference for high-fat foods. A score of 0 denoted no preference for either food type. The details of the questionnaire are presented in Table S1.

### Animals and treatments

The animal study was reviewed and approved by the Animal Ethical and Welfare Committee of the Shanghai Jiao Tong University School of Medicine, Affiliated Sixth People’s Hospital. Three-week-old male C57BL/6J mice, a strain sensitive to high fat feed and easily to be induced obesity, were obtained from the Nanjing Biomedical Research Institute of Nanjing University (Nanjing, China). All mice were housed under standard conditions with ad libitum access to water and food. After 3 weeks of acclimatisation, 50 mice were randomly divided into five groups (*n* = 10 per group): CD (10% kcal from fat); HFD with 60%, 70%, and 75% fat, mainly from cocoa butter (HFD-C, Jiangsu Synergetic Pharmaceutical Bioengineering Co., Ltd, Nanjing, China); and HFD with 60% fat, mainly from lard (HFD-L, Research Diets, Inc., New Brunswick, NJ, USA). The fat content in each diet was defined as the percentage of total calories obtained from fat and 20% of total calories were derived from proteins in all diets. The details of the diets are presented in Table S2.

All mice were fed for 24 weeks. Food intake was calculated every 2–3 days, based on the amount of food given and withdrawn. All mice were weighed weekly. Before euthanasia, the mice were evenly and randomly divided into two groups. One group was sacrificed after 15-hour fasting, and the other group was sacrificed on another day. Under isoflurane anaesthesia, mice were sacrificed by cervical dislocation. Plasma samples were collected separately from each orbit. Liver and brain tissues were extracted and immediately placed in liquid nitrogen. All samples were stored at -80 °C before use. Liver tissue samples were treated with 4% paraformaldehyde for Oil red and PAS staining.

Another eight mice were used for the food choice test after 2 weeks of acclimatisation. Mice were housed in four cages. Adequate amounts of CD and HFD-L were simultaneously administered to each cage for a week. Daily intake of the two diets was recorded.

### Blood analysis

Blood glucose and β-hydroxybutyric acid (β-HB) were measured from the tail vein using a glucometer (Roche, Basel, Switzerland) and a blood glucose and ketone test meter (Abbott Laboratories, Chicago, IL, USA), respectively. Serum insulin concentrations were determined using an enzyme-linked immunosorbent assay kit (Crystal Chem; Cook, IL, USA). The homeostatic model assessment for insulin resistance (HOMA-IR) was calculated as: (fasting glucose in mmol/L × fasting insulin, µU/mL) / 22.5. Serum alanine transaminase (ALT), total cholesterol (TC), TG, high-density lipoprotein (HDL), low-density lipoprotein (LDL), and free fatty acid (FFA) levels were determined using an autoanalyzer (Hitachi Inc., Tokyo, Japan). Liver glycogen levels were determined using quantitative colorimetric assay kits (Bioassay Systems, Hayward, CA, USA).

### Histological examinations

For Oil Red O staining, liver tissue samples were fixed and embedded in optimal cutting temperature compounds after sucrose dehydration. Standard frozen sections, 8 μm in thickness, were obtained. Sections were stained with Oil Red O solution for 10 min, rinsed with distilled water, differentiated with 75% alcohol, and counterstained with haematoxylin. For periodic acid-Schiff (PAS) staining, the specimens were embedded after dehydration using an ethanol gradient. Liver tissues were cut into 4 μm thick sections. After dewaxing and oxidation, the sections were stained with Schiff reagent for 20–25 min and counterstained with haematoxylin.

### Tolerance test

Intraperitoneal glucose tolerance test (IPGTT), pyruvate tolerance test (PTT), and insulin tolerance test (ITT) are classic tests used to assess overall glucose metabolism, gluconeogenesis, and insulin sensitivity, respectively. For the IPGTT and PTT, the mice were fasted for 12 h and then intraperitoneally injected with D-glucose or pyruvate at a dose of 2 g/kg. For the ITT, the mice were fasted for 6 h and then injected intraperitoneally with insulin (1 U/kg). Blood glucose levels were measured 0, 15, 30, 60, and 120 min after injection. The area under the curve (AUC) was calculated using a trapezoidal formula.

### Quantitative real-time PCR (qPCR)

TRIzol reagent (Thermo Scientific, Waltham, MA, USA) was used to extract total RNA from the hypothalamic tissues. The sequences of the primers used for qPCR were as follows: Npy: forward 5’-ATGCTAGGTAACAAGCGAATGG-3’, reverse 5’-TGTCGCAGAGCGGAGTAGTAT-3’; Agrp: forward 5’-AGAGTTCCCAGGTCTAAGTCTG-3’, reverse 5’-GCGGTTCTGTGGATCTAGCA-3’; Pomc: forward 5’-ATGCCGAGATTCTGCTACAGT-3’, reverse 5’-CCACACATCTATGGAGGTCTGAA-3’; Cartpt: forward 5’-CCCGAGCCCTG-GACATCTA-3’, reverse 5’-GCTTCGATCTGCAACATAGCG-3’; and glyceraldehyde 3-phosphate dehydrogenase (Gapdh): forward 5’-AGGTCGGTGTGAACGGATTTG-3’, reverse 5’-GGGGTCGTTGATGGCAACA-3’.

### Statistical analyses

All statistical analyses were performed using SPSS software (version 22.0; IBM Corp., Armonk, NY, USA). Results are presented as mean ± standard deviation (SD). Macronutrient content was expressed as a percentage of the total energy intake. The Shapiro–Wilk test was used to check for normality. Differences among groups were analysed using analysis of variance. Paired-samples *t* test was used to examine within-group differences and independent-samples *t* tests were used to explore between-group differences.

## Results

### VLCD group had greater weight loss and better glycaemic control

**Fig. 1:**
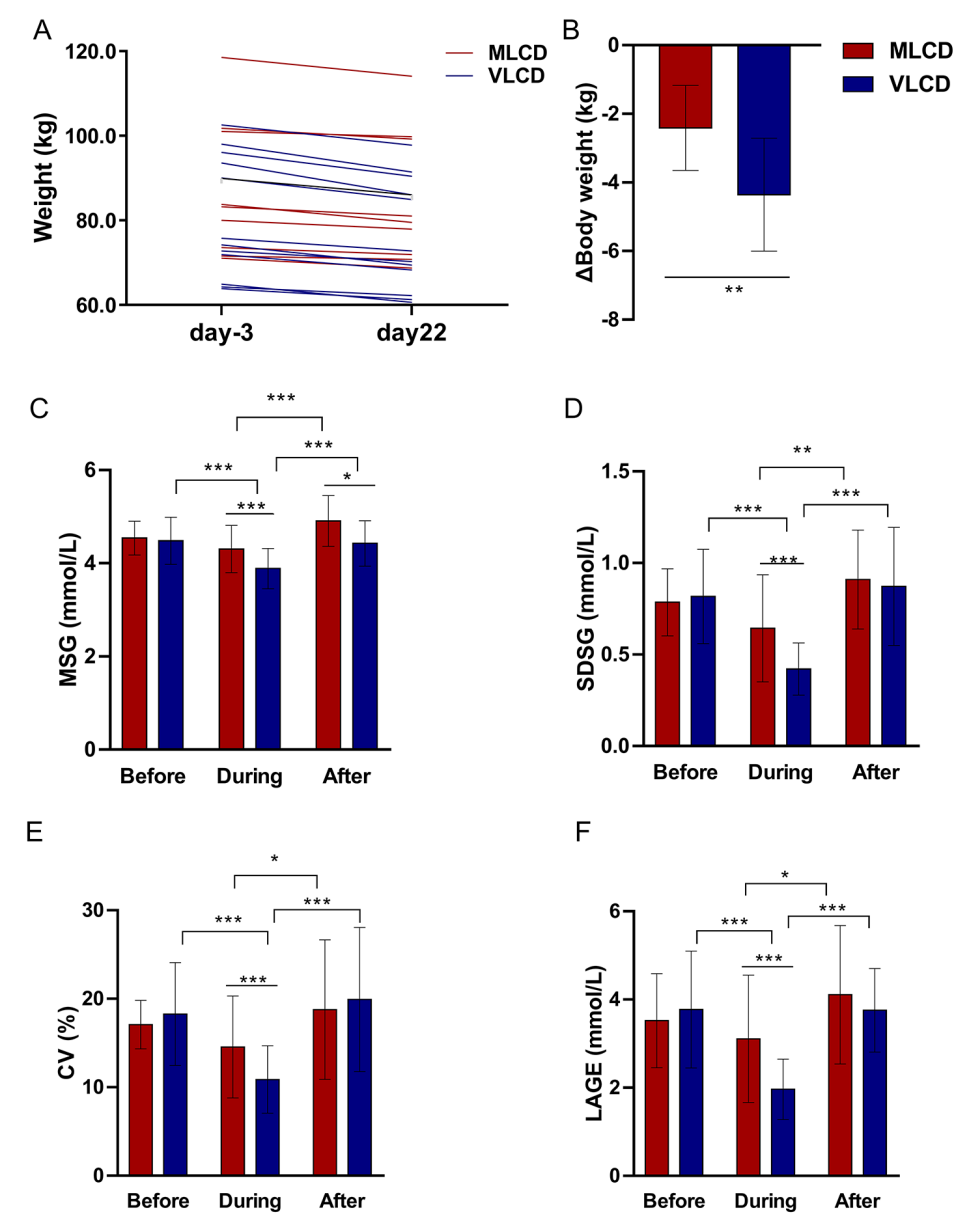
Body weight and blood glucose response to LCD in human study. (**A**) Body weight trend during the intervention. (**B**) Changes in body weight. Curves of (**C**) MSG, (**D**) SDSG, (**E**) CV, and (**F**) LAGE Notes: Data are expressed as mean ± SD (n = 9 for the MLCD group and n = 13 for the VLCD group). Independent-samples *t* test was used. * *P* < 0.05; ** *P* < 0.01; *** *P* < 0.001 Abbreviations: MLCD: moderately low-carbohydrate diet; VLCD: very low-carbohydrate diet; Before: 3 days before the intervention; During: during the intervention (21 days); After: 3 days after the intervention; MSG: mean sensor glucose; SDSG: standard deviations of sensor glucose; CV: coefficient of variation; LAGE: largest amplitude of glycemic excursion

After the 3-week intervention, significant weight loss was evident in both the MLCD and VLCD groups (*P* < 0.001, Fig. 1A). Body weight decreased by 2.41 ± 1.20 kg (2.7%) in the MLCD group and 4.35 ± 1.60 kg (5.3%) in the VLCD group (Fig. 1B). The loss in the VLCD group was significantly greater than that in the MLCD group (*P* < 0.01). The mean glucose levels and glycaemic fluctuations before and during the intervention were compared, and the MLCD group showed a trend of reduction, but the difference was not statistically significant, whereas the VLCD group showed a significant decrease (Fig. 1C-F). The VLCD group had significantly lower values than the MLCD group for MSG (4.30 ± 0.06 vs. 3.89 ± 0.03 mmol/L), SDSG (0.64 ± 0.03 vs. 0.42 ± 0.01 mmol/L), CV (14.37 ± 0.65% vs. 10.87 ± 0.31%) and LAGE (3.11 ± 0.16 vs. 1.96 ± 0.06 mmol/L) (all *P* < 0.001). After day 21, the subjects returned to their former diet for 3 days. The favourable effects on glycaemic control disappeared. Blood glucose levels and glycaemic swings returned to baseline in both groups.

### Dietary patterns and food preferences of the subjects

**Fig. 2:**
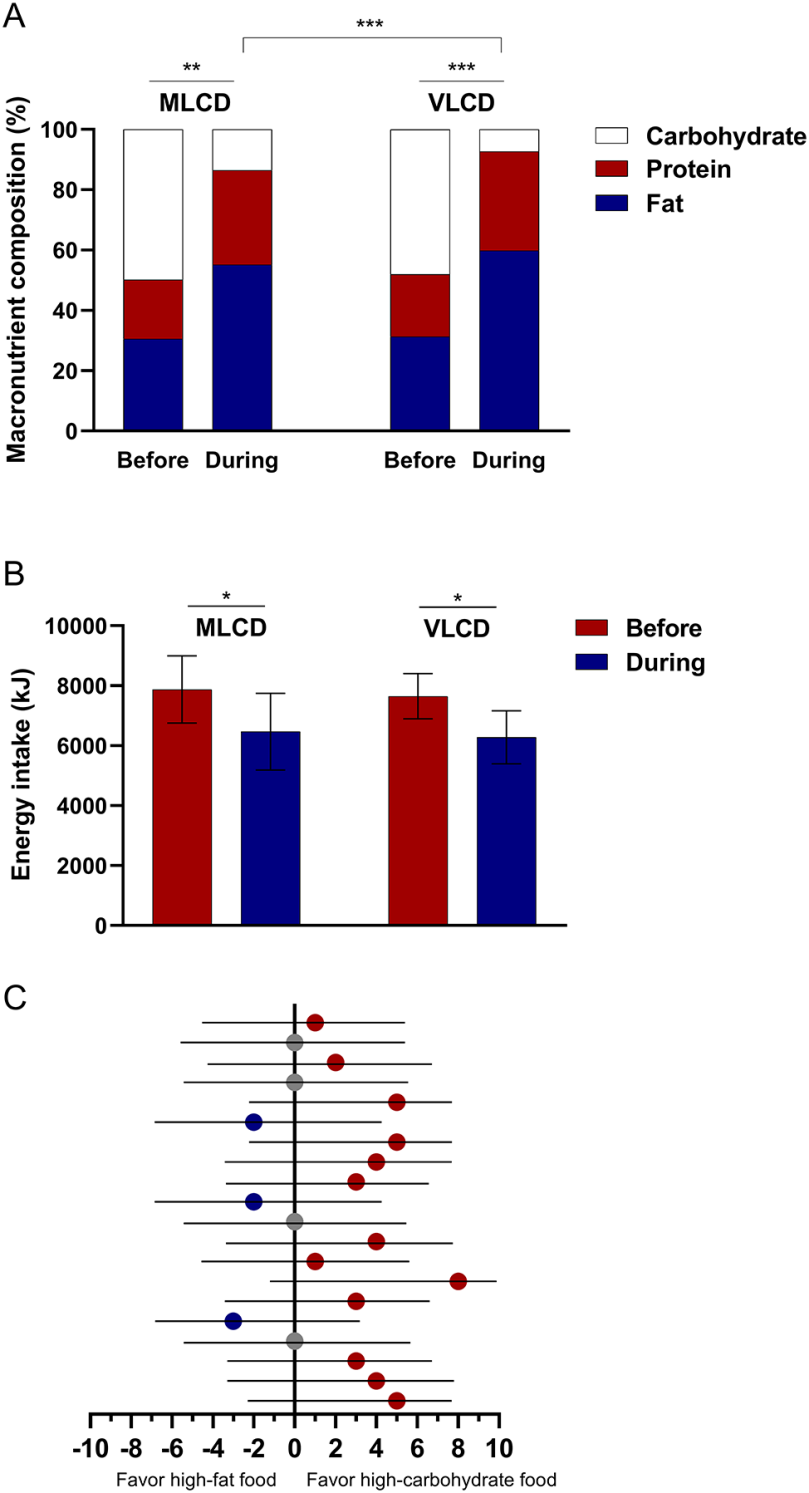
Macronutrient compositions of diets and surveyed food preferences in subjects. (**A**) Macronutrient compositions of diets. (**B**) Total energy intake. (**C**) Total scores of the subjects Notes: Data are expressed as mean ± SD and the macronutrient content is expressed as a percentage of total energy intake (n = 9 for the MLCD group and n = 13 for the VLCD group). Independent-samples *t* test and paired *t* test were used. * *P* < 0.05; ** *P* < 0.01; *** *P* < 0.001. Figure 2C: Dots on the left side of the vertical axis indicate preference for high-fat foods. Dots on the right side of the vertical axis indicate preference for high-carbohydrate foods. Dots on the vertical axis indicate no preference for either Abbreviations: MLCD: moderately low-carbohydrate diet; VLCD: very low-carbohydrate diet; Before: 3 days before the intervention; During: during the intervention (21 days)

Before adopting the LCD, all the subjects consumed a normal diet. The percentage of fat, protein, and carbohydrate in the pre-intervention diet was 30.50 ± 3.07%, 19.68 ± 1.58%, 49.82 ± 4.08% in the MLCD group, and 31.27 ± 3.03%, 20.67 ± 3.33%, 48.05 ± 3.87% in the VLCD group, respectively. There were no statistically significant differences between the two groups. During the intervention, the macronutrient compositions of fat, protein, and carbohydrate changed to 55.15 ± 2.61%, 31.28 ± 3.38%, 13.57 ± 2.18% in the MLCD group, and 59.79 ± 5.33%, 32.89 ± 4.77%, 7.32 ± 2.36% in the VLCD group, respectively (Fig. 2A). Carbohydrate intake in the VLCD group was approximately 48% lower than that in the MLCD group (*P* < 0.001). During the intervention, daily calorie intake significantly decreased in both groups (all *P* < 0.05; Fig. 2B).

Twenty participants completed the food preference survey. Of these, 13 preferred high-carbohydrate foods, three favoured high-fat foods, and four showed no preference (Fig. 2C). These findings revealed an overall preference for high-carbohydrate foods.

### HFD-related metabolic disorders in mice

**Fig. 3:**
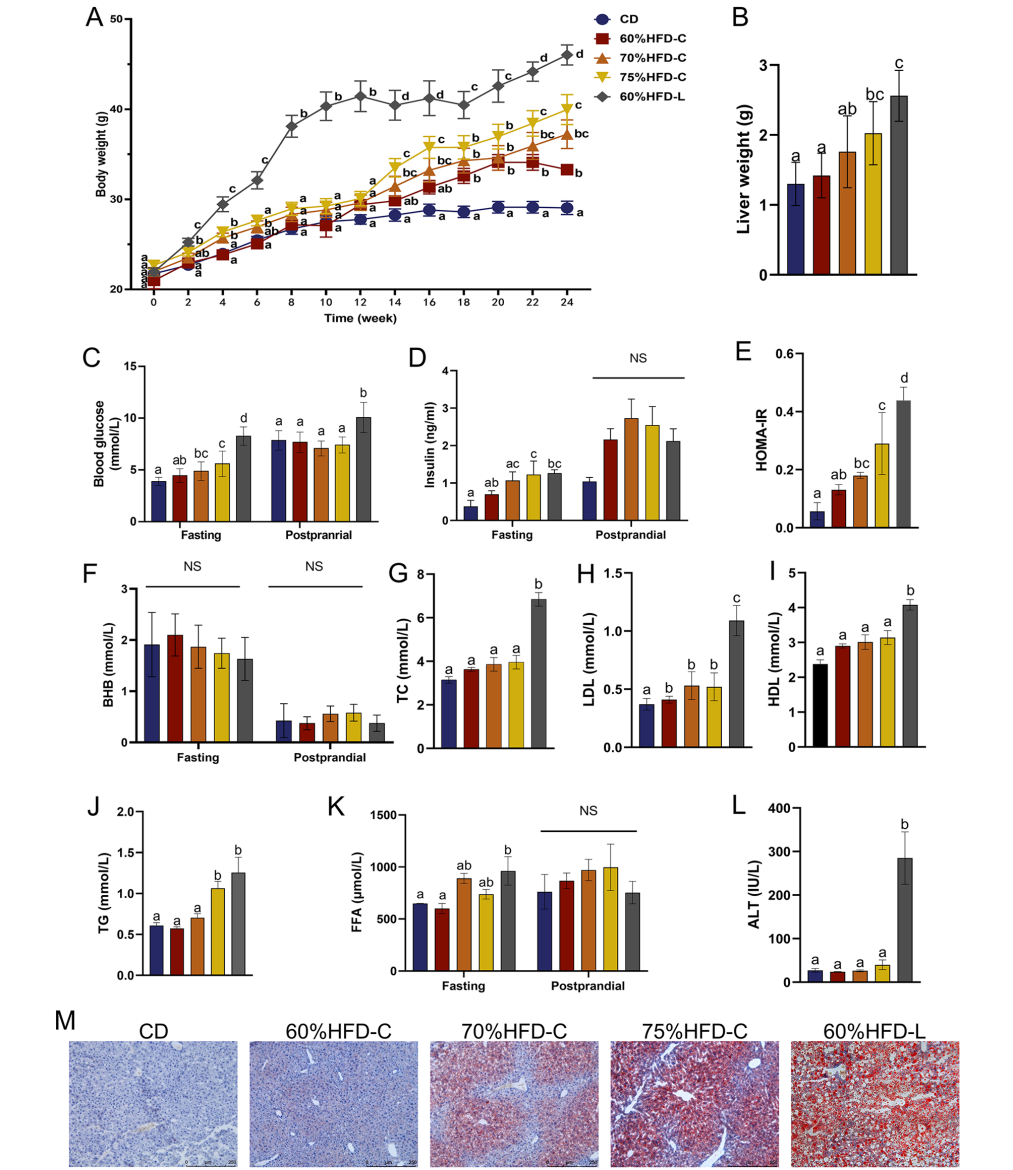
Effects of different diets on body weight and glucolipid metabolism in mice. Panels A-M respectively show values of body weight, liver weight, blood glucose, insulin, HOMA-IR, blood β-HB, fasting serum TC, LDL, HDL, TG, serum FFA, fasting serum ALT, and Oil red O staining of liver tissue Notes: Data are expressed as mean ± SD (n = 10 for A & C-F and n = 5 for B & G-L). Analysis of Variance and independent-samples *t* test were used. If the letters on the top of bars are different it means there is statistical difference between the two groups (*P* < 0.05); NS, no statistical difference Abbreviations: CD: chow diet; 60%HFD-C: HFD with 60% fat mainly from cocoa butter; 70%HFD-C: HFD with 70% fat mainly from cocoa butter; 75%HFD-C: HFD with 75% fat mainly from cocoa butter. 60%HFD-L: HFD with 60% fat mainly from lard; HOMA-IR: homeostatic model assessment for insulin resistance; TC: total cholesterol; LDL: low-density lipoprotein; HDL: high-density lipo-protein; TG: triglyceride; FFA: free fatty acid; β-HB: β-hydroxybutyric acid; ALT: Serum alanine transaminase

After 24 weeks of feeding, the HFD groups showed higher body and liver weights than the CD group (all *P* < 0.001, Fig. 3A and B). Fasting blood glucose, fasting insulin, and HOMA-IR were also higher in the HFD group than in the CD group, revealing impaired glucose metabolism and decreased insulin sensitivity in HFD-fed mice (all *P* < 0.05, Fig. 3C-E). The levels of blood β-HB did not differ significantly among the five groups (Fig. 3F). A significant increase in hepatic lipid deposition in HFD-fed mice was observed by Oil Red O staining compared to CD mice (Fig. 3M).

Among the three HFD-C groups, increasing dietary fat content was associated with increased weight gain, higher fasting blood glucose levels, more severely decreased insulin sensitivity, and greater hepatic lipid accumulation in mice (Fig. 3A, C, E, and M). Body weight, blood glucose, HOMA-IR, TC, LDL, HDL, TG, FFA, and liver lipid deposition were higher in the HFD-L group than those in the HFD-C and CD groups (Fig. 3A, C, E, G-K, and M). The serum ALT concentration was also significantly elevated in the HFD-L group, indicating impaired liver function (Fig. 3L).

### Impaired glucose tolerance, decreased insulin sensitivity, and hyper-gluconeogenesis in HFD-fed mice

**Fig. 4:**
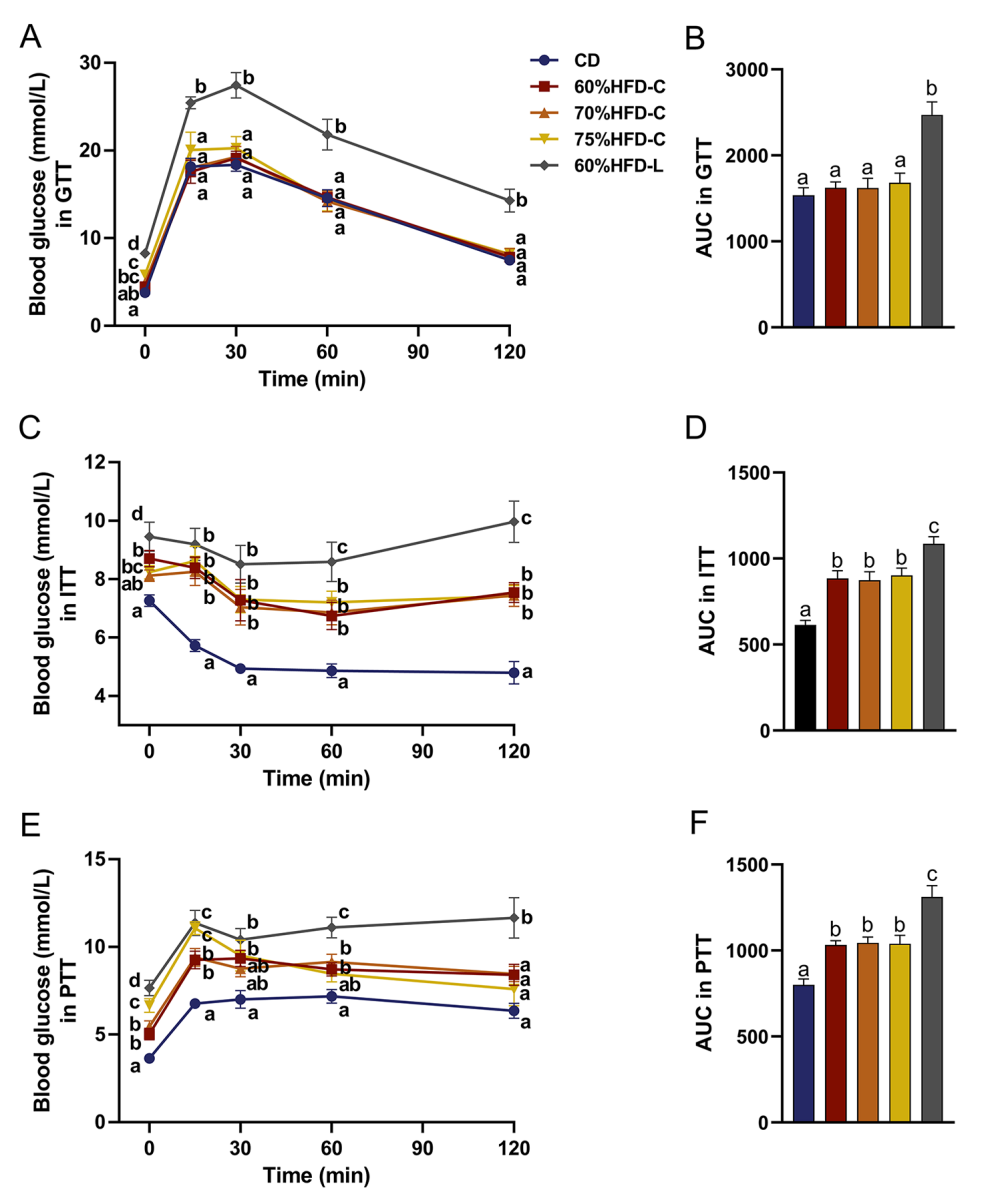
Tolerance tests in mice fed with different diets. Curves and AUCs for (**A**) IPGTT, (**B**) ITT, and (**C**) PTT Notes: Data are expressed as mean ± SD (n = 10). Analysis of Variance and independent-samples *t* test were used. If the letters on the top of or beside bars are different it means there is statistical difference between the two groups (*P* < 0.05) Abbreviations: CD: chow diet; 60%HFD-C: HFD with 60% fat mainly from cocoa butter; 70%HFD-C: HFD with 70% fat mainly from cocoa butter; 75%HFD-C: HFD with 75% fat mainly from cocoa butter. 60%HFD-L: HFD with 60% fat mainly from lard; AUC: area under the curve; IPGTT: intraperitoneal glucose tolerance test; ITT: insulin tolerance test; PTT: pyruvate tolerance test

In the IPGTT, the blood glucose levels of the HFD-L group were always significantly higher than those of the other groups (all *P* < 0.01; Fig. 4A). The AUC in the HFD-L group was also much higher, indicating impaired glucose tolerance (*P* < 0.001; Fig. 4B). In the ITT, after the administration of insulin, all HFD groups presented higher blood glucose levels at all time points and greater AUCs than the corresponding values in the CD group (*P* < 0.01; Fig. 4C and D). Sixty minutes after insulin injection, the blood glucose levels of lard-fed mice were significantly higher than those of cocoa butter-fed mice. Compared with the other groups, the HFD-L group displayed a higher AUC. This finding indicated that HFD reduced insulin sensitivity in mice, and lard aggravated this resistance compared to cocoa butter. In the PTT, compared with CD-fed mice, HFD-fed mice showed higher glycaemia in response to the administration of pyruvate, a substrate of gluconeogenesis (Fig. 4E). The AUCs were greater for mice in the HFD groups compared with that for mice in the CD group, with the highest value in the HFD-L group (*P* < 0.001, Fig. 4F). These findings indicated that HFD upregulated hepatic gluconeogenesis, with the effect being more significant in lard-fed mice.

### Preference for HFD by mice

**Fig. 5:**
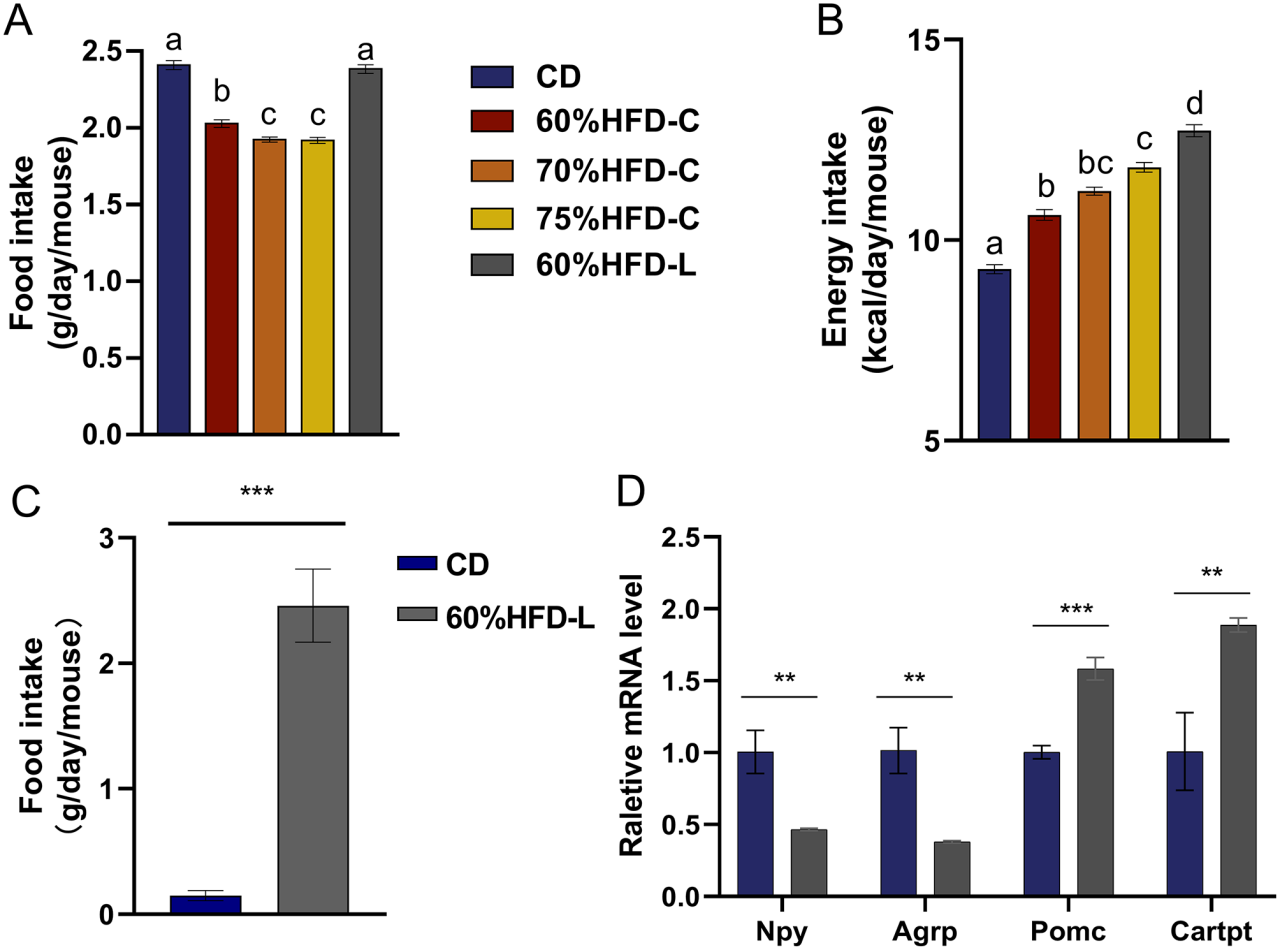
Food intake, energy intake, and mRNA levels of hypothalamic hormones in mice. (**A**) Food intake. (**B**) Energy intake. (**C**) Food intake of mice provided with CD and HFD-L simultaneously. (**D**) The mRNA levels of Agrp, Npy, Pomc, and Cartpt in hypothalamus Notes: Data are expressed as mean ± SD (n = 10 for A& B, n = 8 for C and n = 5 for D). Analysis of Variance and independent-samples *t* test were used. If the letters on the top of bars are different mean there are statistical difference between the two groups (*P* < 0.05). * *P* < 0.05; ** *P* < 0.01; *** *P* < 0.001

The total food intake in the HFD-C group was lower than that in the CD and HFD-L groups (both *P* < 0.05; Fig. 5A). However, energy intake in the HFD group was significantly greater than that in the CD group (*P* < 0.001, Fig. 5B). Mice in the HFD-C group consumed more calories as the dietary fat content increased. Lard-fed mice consumed more energy than cocoa butter-fed mice. When provided with CD and HFD-L simultaneously, mice almost exclusively consumed HFD-L (0.15 ± 0.04 vs. 2.46 ± 0.29 g/day/mouse, *P* < 0.001, Fig. 5C). The mice clearly preferred HFD over CD. qPCR revealed decreased mRNA levels of Npy and Agrp (both *P* < 0.01) and increased mRNA levels of Pomc and Cartpt (both *P* < 0.001) in the hypothalamus of HFD-fed mice compared to CD-fed mice (Fig. 5D). Npy and Agrp are hypothalamic orexigenic genes, whereas Pomc and Cartpt are hypothalamic anorexigenic genes [22–26]. The HFD-fed mice were in a relatively satiated state after overnight fasting owing to excessive energy intake.

### Liver glycogen content is not decreased in HFD-fed mice during fasting

**Fig. 6:**
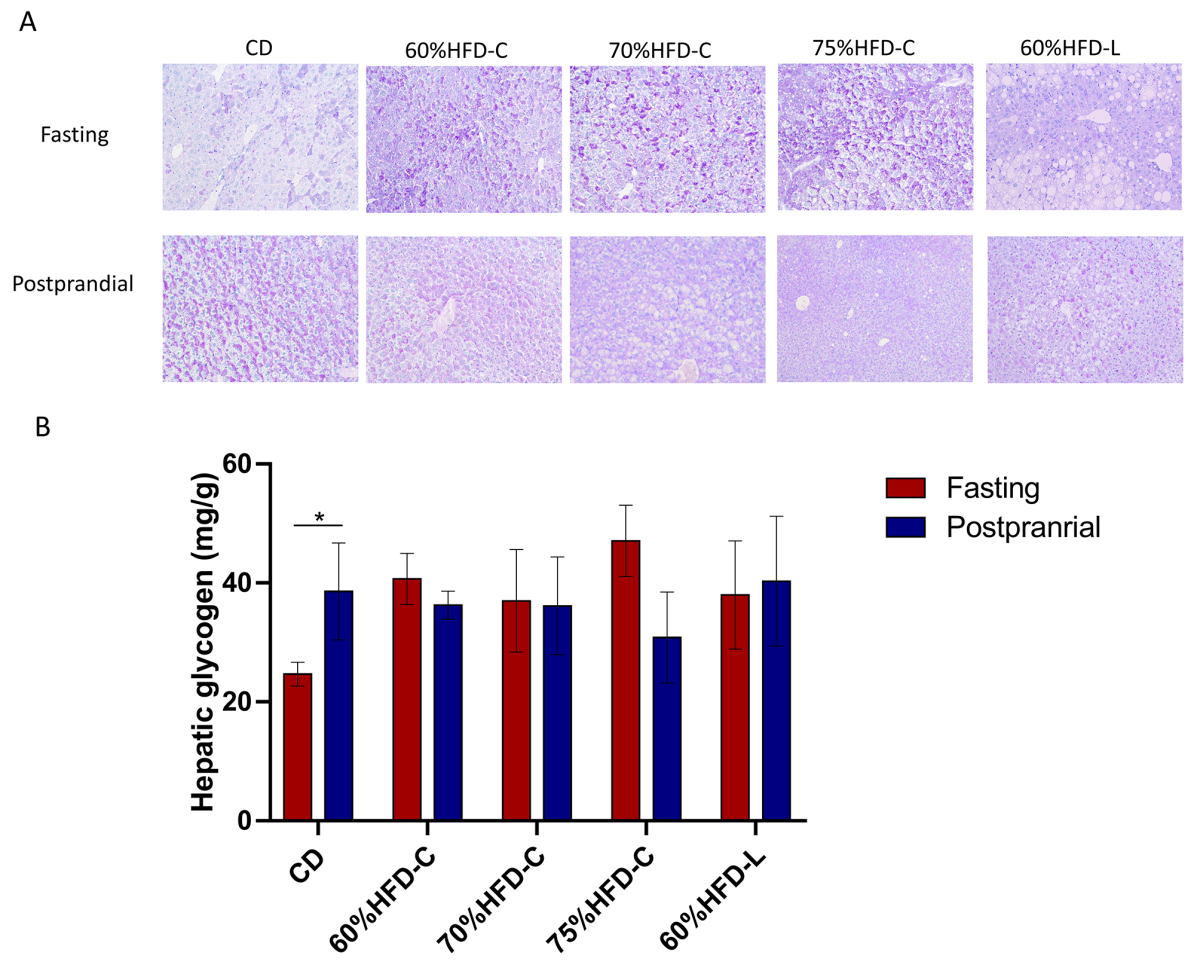
Hepatic glycogen in mice. (**A**) PAS staining in liver tissue. (**B**) Glycogen level in liver tissue Notes: Data are expressed as mean ± SD (n = 5). Paired *t* test was used. * *P* < 0.05

PAS staining and glycogen measurement revealed that the fasting liver glycogen content was approximately 36% lower than the postprandial level in the CD group (*P* < 0.05; Fig. 6A and B). However, there were no significant differences between the fasting and postprandial hepatic glycogen levels in the HFD groups. In the fasting state, hepatic glycogen levels were higher in the HFD groups than in the CD group (All *P* < 0.05). No differences were observed among the HFD groups. There were no differences in the postprandial state among the five groups.

## Discussion

Previous studies on LCDs have primarily focused on comparing them to moderate carbohydrate diets (about 30–50% carbohydrate) [27–29] or low-fat diets [7, 30]. Few studies, however, have explored the differences between MLCD and VLCD. In particular, whether lower carbohydrate intake (e.g., < 10%) will bring about greater improvement in metabolism is controversial when carbohydrate intake is already < 20% [7, 31]. This study revealed the greater efficacy of VLCD in weight loss and glucose homeostasis. During the intervention, the VLCD group consumed approximately 52% less carbohydrates than the MLCD group, while no significant difference in total calorie intake was observed between the two groups. Although both groups experienced reductions in body weight, the VLCD group exhibited a roughly twofold greater decrease compared to the MLCD group, implying a promising effect in weight control. However, a meta-analysis that included 50 trials indicated a U-shaped effect of carbohydrate restriction on body weight, with the most significant reduction observed in the 35% carbohydrate diet [30]. When carbohydrate intake was < 35%, body weight slightly increased with decreasing proportions of carbohydrates in diets over a relatively long-term observation, probably due to the challenges of adhering to a strict LCD for a long time. Therefore, balance between the amount of daily carbohydrate intake and the possibility of adhering to it over an extended period is crucial to weight management. To the best of our knowledge, no studies have directly compared the effects of MLCD (10–20% carbohydrate) and VLCD (≤ 10% carbohydrate), hence, future studies are needed to provide a more comprehensive understanding of the difference between MLCD and VLCD.

Given the exclusion of individuals with diabetes from this study, significant reductions in blood glucose levels and fluctuations were only observed when carbohydrate intake was restricted to 10%, as seen in the VLCD group, rather than in the MLCD group. Reducing glucose fluctuation is important for patients with chronic conditions, such as diabetes, cardiovascular diseases, and obesity, because decreased glycaemic excursions are beneficial for improving insulin sensitivity, oxidative stress, and endothelial dysfunction [32, 33]. High glucose variability is a critical predictor of macrovascular and microvascular complications [34, 35]. In addition, severe glucose variability, usually in the form of glucose spikes, is also observed in individuals with normoglycemia and is associated with a high risk of developing diabetes [36]. VLCD can effectively lower glucose swings and may reduce the risk of diabetes and its vascular complications.

The original intention of this study was to replicate the effect of LCDs on mice and reveal the underlying mechanism. However, the effects of LCD/HFD appeared to diverge between humans and mice, and different food preference was considered as the critical factor. Specifically, in mice, dietary fat seemed to stimulate energy intake, whereas in humans, it had the opposite effect, suppressing appetite and promoting sense of fullness and satiety. Both the MLCD and VLCD groups exhibited a significant reduction in total energy intake, implying that the favourable metabolic outcomes associated with LCDs might be partially attributed to calorie reduction. In contrast, when food with different fat content was provided ad libitum, mice preferred the HFD and consumed more calories. In animals, food intake is influenced by a balance between homeostatic control and hedonic values. However, this equilibrium was disrupted in the mice on HFD. Dietary fat stimulates the hedonic systems that override internal homeostatic mechanisms and promote energy intake [37]. In the fasting state, mice in the HFD group showed lower expression of starvation-related genes and higher expression of satiety-related genes than mice in the CD group. This suggested that, even after an overnight fasting, HFD-fed mice remained relatively energy-replete due to their excess food consumption compared with CD-fed mice. This excessive calorie intake accounts for why the HFD-fed mice failed to replicate the effects induced by LCDs in humans.

The study further revealed that the dietary fat source and fat content could also influence energy intake in mice. Calorie intake in the HFD-L group was higher than that of the HFD-C group, and even exceeded that of the HFD-C group with 75% fat content. This preference for lard over cocoa butter by mice led to an accumulation of excess energy and subsequent metabolic deterioration. Preference for lard in mice has rarely been reported. In contrast, humans usually favour cocoa butter over lard. Furthermore, when the fat content varied for the same fat source, a distinct positive correlation between fat content and total energy intake in mice emerged. A similar result was observed that fat intake increased significantly with increasing fat content, thereby contributing to higher energy intake and, consequently, weight gain [37]. This phenomenon can be explained by hypothalamic hunger pathways, including 5-HT receptors, and the dopamine and opioid signalling pathways stimulated by fat instead of protein or sucrose [37].

It is also worth noting that the protein content in the HFD for mice differed from that in humans. The reasons for applying a 20% protein content in HFD of mice can be summarised in the following three aspects. Firstly, the widely adopted standard CD for mice comprises 20% protein of total calories. Secondly, a higher protein proportion, such as 30%, would have been unfeasible due to the study’s objective of investigating HFDs with a fat content exceeding 70%. Most importantly, despite the satiating effect of a high-protein diet, typically around 35% [38], a previous study indicated that protein content did not influence total energy intake in mice [37]. Another aspect worth mentioning regarding the design of animal experiment is the choice of the C57BL/6 strain. C57BL/6 mice are the most widely used strain in studies of metabolism worldwide, readily available and with relatively better cost performance. Other common stains, such as Kunming, ICR, and BALB/c, are much more commonly used in research related to immunology and oncology, even though they have shown similar responses to HFD as the C57BL/6 strain [39]. Another strain, ob/ob mice, is characterized by a mutation in the leptin gene, resulting in hyperphagic and overeating due to the absence of functional leptin [40]. Thus, they fail to represent the natural physiological status and are not suitable for our study as well.

In addition, liver glycogen content at both fasting and postprandial levels did not differ in HFD-fed mice in this study, which is consistent with the authors’ previous findings [41]. The elevated levels of fasting insulin in HFD-fed mice might be one of the reasons for this, as increased insulin levels are known to stimulate glycogen synthesis. Furthermore, hyperglycaemia inhibits glycogen breakdown. Combined with the PTT results, it can be speculated that fasting energy utilisation in HFD-fed mice predominantly relies on lipolysis and hypergluconeogenesis, rather than glycogenolysis. In CD-fed mice, the fasting liver glycogen content was much lower than the postprandial levels, suggesting that glycogen may serve as one of the primary energy sources during fasting.

### Study strengths and limitations

Until now, few studies have investigated the differences in food preferences between humans and mice or analysed the mechanisms underlying this inconsistency. This study also had several limitations. First, the sample size of the clinical trial was small which might have augmented the potential for bias and could have influenced the robustness of the results. Second, the follow-up duration was relatively brief, and whether the VLCD-induced effects could be sustained for a long time remains unknown. Third, this study was a retrospective subgroup analysis of a previous clinical trial. Therefore, larger randomised trials with longer follow-up periods are needed to ascertain the long-term effects of MLCD and VLCD. In addition, the protein contents in the diets of human and animal study were not exactly the same as aforementioned, although a previous study has confirmed that protein content did not influence the total energy intake in mice. Future attention should be given to this issue, and more precise and well-matched animal studies are warranted for further mechanistic investigations.

## Conclusion

This study indicates that lower carbohydrate intake (such as carbohydrate intake ≤ 10%) can contribute to decreased energy intake and better metabolic improvement in individuals with obesity. However, diets with lower carbohydrates and higher fat contents led to increased energy intake and deterioration in metabolism in mice. Different food preferences, in that human prefer high-carbohydrate foods and mice prefer high-fat foods might be the main reason for the contrasting results between humans and mice. This study raises awareness that it is not what you eat but how much you eat that matters in weight control. Preferred foods are easy to overindulge in, and restricting intake is crucial for energy balance and weight management.

### Electronic supplementary material

Below is the link to the electronic supplementary material.

Supplementary Material 1

## Data Availability

The datasets used and/or analysed during the current study are available from the corresponding author on reasonable request.

## Abbreviations

ALT: Alanine transaminase
AUC: Area under the curve
β-HB: β-hydroxybutyric acid
CD: Chow diet
CV: Coefficient of variance
FFA: Free fatty acid
HDL: High density lipoprotein
HFD: High-fat diet
HFD-C: High-fat diet cocoa butter
HFD-L: High-fat diet lard
HOMA-IR: Homeostatic model assessment for insulin resistance
IPGTT: Intraperitoneal glucose tolerance test
ITT: Insulin tolerance test
LAGE: Largest amplitude of glycaemic excursion
LCD: Low-carbohydrate diet
LDL: Low density lipoprotein
MLCD: Moderate low-carbohydrate diet
MSG: Mean sensor glucose
MUFA: Monounsaturated fatty acids
PAS: Perodic acid-Schiff
PTT: Pyruvate tolerance test
PUFA: Polyunsaturated fatty acids
SD: Standard deviation
SDSG: Standard deviations of sensor glucose
SFA: Saturated fatty acids
TC: Total cholesterol
TG: Triglyceride
VLCD: Very low-carbohydrate diet
