# Supplementary material for: Opposite effects of low-carbohydrate high-fat diet on metabolism in humans and mice
Source: Lipids Health Dis. 2023 Nov 10;22:191. doi: 10.1186/s12944-023-01956-3 (PMC10636972; doi:10.1186/s12944-023-01956-3)
Supplement: Supplementary file 1 — Supplementary Material 1 [file 12944_2023_1956_MOESM1_ESM.docx]

| Table S1. Dietary macronutrient compositions of foods provided in food preference survey questionnaire | | | | |
| --- | --- | --- | --- | --- |
|  |  | protein | fat | carbohydrate |
|  |  | kcal% | kcal% | kcal% |
| Q1 |  |  |  |  |
|  | multi flavor peanuts | 2.93 | 16.98 | 80.09 |
|  | fried peanuts | 14.81 | 74.79 | 10.40 |
| Q2 |  |  |  |  |
|  | pork steamed buns | 25.80 | 22.01 | 52.19 |
|  | meatballs | 22.21 | 60.08 | 17.71 |
| Q3 |  |  |  |  |
|  | cheese cake | 11.86 | 31.79 | 56.35 |
|  | cream cheese | 31.31 | 64.42 | 4.26 |
| Q4 |  |  |  |  |
|  | boiled eggs | 37.59 | 55.52 | 6.89 |
|  | egg yolk puff pastries | 12.02 | 9.01 | 78.97 |
| Q5 |  |  |  |  |
|  | apples | 2.75 | 3.09 | 94.16 |
|  | avocados | 4.56 | 78.55 | 16.89 |
| Q6 |  |  |  |  |
|  | flavored yoghurts | 13.60 | 31.58 | 54.82 |
|  | sugar-free yogurts | 20.39 | 52.43 | 27.18 |
| Q7 |  |  |  |  |
|  | sandwiches | 11.52 | 27.03 | 61.45 |
|  | ham slices | 19.38 | 74.68 | 5.94 |
| Q8 |  |  |  |  |
|  | roast duck | 23.59 | 66.47 | 9.94 |
|  | roast duck with pancakes | 16.59 | 32.04 | 51.37 |
| Q9 |  |  |  |  |
|  | roasted sausages | 19.26 | 60.00 | 20.74 |
|  | pizza | 18.47 | 28.63 | 52.90 |
|  | mozzarella | 31.31 | 64.42 | 4.26 |
| Q10 |  |  |  |  |
|  | milk tea | 2.12 | 53.59 | 44.29 |
|  | bubble tea | 2.24 | 8.44 | 89.33 |

| Table S2. Formulations of diets | | | | | | | | | | |
| --- | --- | --- | --- | --- | --- | --- | --- | --- | --- | --- |
| Product | CD | | 60%HFD-C | | 70%HFD-C | | 75%HFD-C | | 60%HFD-L | |
|  | gm | Kcal | gm | Kcal | gm | Kcal | gm | Kcal | gm | Kcal |
| Protein | 200 | 800 | 260 | 1040 | 300 | 1200 | 310 | 1240 | 260 | 1040 |
| Carbohydrate | 670 | 2680 | 260 | 1040 | 150 | 600 | 80 | 320 | 260 | 1040 |
| Fat | 40 | 360 | 350 | 3150 | 460 | 4140 | 520 | 4680 | 350 | 3150 |
| Ingredient |  |  |  |  |  |  |  |  |  |  |
| Casein | 200.00 | 800.00 | 262.00 | 1048.00 | 301.30 | 1205.20 | 310.47 | 1241.88 | 258.00 | 1032.00 |
| L-Cystine | 3.00 | 7.20 | 3.93 | 15.72 | 3.93 | 15.72 | 3.93 | 15.72 | 3.87 | 15.48 |
| Corn Starch | 427.00 | 1708.00 |  |  |  |  |  |  |  |  |
| Maltodextrin 10 | 116.00 | 464.00 | 127.07 | 508.28 | 55.02 | 220.08 | 17.03 | 68.12 | 161.25 | 645.00 |
| Sucrose | 116.00 | 464.00 | 127.07 | 508.28 | 55.02 | 220.08 | 17.03 | 68.12 | 88.75 | 355.01 |
| Cellulose, BW200 | 50.00 | 0.00 | 65.50 | 0.00 | 65.50 | 0.00 | 65.50 | 0.00 | 64.50 | 0.00 |
| Soybean Oil | 40.00 | 360.00 | 52.40 | 471.60 | 52.40 | 471.60 | 52.40 | 471.60 | 32.25 | 290.25 |
| Lard |  |  |  |  |  |  |  |  | 316.05 | 2844.45 |
| Cocoa Butter |  |  | 301.30 | 2711.70 | 406.10 | 3654.90 | 471.60 | 4244.40 |  |  |
| Mineral Mix | 35.00 | 0.00 | 45.85 | 0.00 | 45.85 | 0.00 | 45.85 | 0.00 | 58.05 | 0.00 |
| Vitamin Mix | 10.00 | 40.00 | 13.10 | 52.40 | 13.10 | 52.40 | 13.10 | 52.40 | 12.90 | 51.60 |
| Choline Bitartrate | 2.50 | 0.00 | 3.28 | 0.00 | 3.28 | 0.00 | 3.28 | 0.00 | 2.58 | 0.00 |
| FD&C Blue Dye #1 |  |  |  |  |  |  |  |  | 0.05 | 0 |
| Total | 1000.00 | 3850.00 | 1000.00 | 5240.00 | 1000.00 | 5940.00 | 1000.00 | 6240.00 | 1000.00 | 5240.00 |
